# No Incidental Memory Advantage for Mixed Handed vs. Consistent Right Handed Participants: Conflicting Results From Earlier Research

**DOI:** 10.1177/00315125241291266

**Published:** 2024-10-12

**Authors:** Henriette Johansen, Emilie H. Rusten, René Westerhausen

**Affiliations:** Department of Psychology, 6305 University of Oslo, Oslo, Norway

**Keywords:** handedness, incidental memory, laterality, learning and memory, hand preference

## Abstract

Individuals who vary their preferred hand when performing different types of manual activities, so-called mixed handers (MH), have been frequently reported to outperform individuals with a consistent (right) hand preference (cRH) on tasks assessing declarative-memory functions. For example, in one influential study, this MH advantage extended to incidental learning from presumed “deep” semantic processing of verbal stimuli but not from “shallow” phonemic or structural processing. In the present study, we aimed to replicate this research finding in two separate participant samples. First, in a pre-registered and sample-size planned experiment we confronted 49 participants (23 MH; 26 cRH) with “phonemic” and “semantic” word evaluation tasks (using a within design), followed by a surprise delayed recognition test. In a second experiment, we repeated the same procedure with 65 other participants (31 MH, 34 cRH). A mixed-effect analyses of variance found a significant main effect of Encoding Condition (phonemic vs. semantic tasks) in both experiments (effect size: *η*_*p*_^*2*^ = .81 to .85), indicating the classical level-of processing effect with higher recognition hits and sensitivity (*d’*) for words that followed semantic versus phonemic encoding. However, the predicted interaction effect of Encoding Condition with Handedness Group was not statistically significant for either sample (all *η*_*p*_^*2*^ < .03), nor was the main effect of Handedness Group. Thus, our findings conflicted with those of the original study in two independent samples. As we had sufficient statistical power to be confident in our failure to detect a genuine group difference, we cannot confirm the previously reported MH over cRH advantage in incidental learning of verbal material. We discuss possible reasons for these contradictory results and the theoretical implications of this discovery.

Functional hemispheric specialization is a core characteristic of human brain organization (Vingerhoets, 2019), and individuals differ in the pattern in which their various cognitive functions are distributed between the cerebral hemispheres (Gerrits et al., 2020; Karlsson et al., 2022). Handedness is arguably the most salient example of this individual diversity. Individuals with a right-hand preference, with left hemispheric dominance for the control of skilled manual actions (e.g., Grabowska et al., 2012; Pool et al., 2014) comprise about 90% of the general population, while those with a left-hand preference constitute about 10% of the general population (Papadatou-Pastou et al., 2020). Definitions of handedness or hand preference have sometimes emphasized the degree, rather than simply the direction, of hand preference (Annett, 1970, 1972; Dragovic & Hammond, 2007). That is, while some individuals consistently prefer the same hand across various manual tasks, others do not show this consistency and switch hands between different tasks (showing a mixed hand preference, MH) or may switch an apparent preference even within the same type of task (showing ambidexterity). This distinction between consistent and mixed-hand preference has led to further research and findings suggesting neuroanatomical differences (e.g., McDowell et al., 2016; Welcome et al., 2009; Witelson, 1989) and differences in cognitive abilities (such as executive functions, mental rotation, divergent thinking) between these groups (e.g., Badzakova-Trajkov et al., 2011; Cheng et al., 2020; Gunstad et al., 2007; Nicholls et al., 2010; Peters & Servos, 1989; Shobe et al., 2009).

One of the best replicated findings of cognitive differences between consistent right handers (cRH) and MH has been a difference in declarative memory ability (for reviews see, Prichard et al., 2023; Prichard et al., 2013). Empirical evidence has accumulated, suggesting a MH advantage for various declarative memory tasks. For example, MH have outperformed cRH in memory recall of word lists after both intentional (e.g., Christman & Butler, 2011; Chu et al., 2012; Propper et al., 2005; Propper et al., 2017) and incidental learning (Christman & Butler, 2011), and irrespective of whether the retrieval task was free recall, cued recall, or recognition judgement (Chu et al., 2012). The MH advantage was found across multiple classical memory phenomena, as reflected in reduced false memory in the Deese-Roediger-McDermott paradigm (Christman et al., 2004), an enhanced source memory (Chu et al., 2012; Lyle, McCabe, & Roediger III, 2008), and improved recall of associate word pairs (e.g., Lyle et al., 2012; Lyle, McCabe, & Roediger III, 2008; Sahu et al., 2016). Furthermore, MH demonstrated more accurate retrieval of autobiographic life events (e.g., Christman et al., 2006; Parker et al., 2017; Propper et al., 2005) and remembered more details in eye-witness testimony paradigms (e.g., Lyle, 2018; Lyle & Jacobs, 2010) or after prose reading (Prichard & Christman, 2017).

The MH advantage has been typically explained by differences in interhemispheric connectivity (e.g., reflected in the size of the corpus callosum) between MH and cRH groups (e.g., Christman & Propper, 2001; Lyle et al., 2017; Prichard et al., 2013). This focus on interhemispheric integration was derived from the Hemispheric Encoding and Retrieval and Asymmetry (HERA) model (R. Habib et al., 2003; Tulving et al., 1994), which observes that encoding of episodic memory leads to higher activation in the left prefrontal cortex while retrieval elicits higher activation in the right prefrontal cortex. Establishing such a functional asymmetry pattern is thought to rely on interhemispheric interaction (Kompus et al., 2011), whereby improved callosal connectivity is associated with improved episodic memory (Putnam et al., 2008). In line with this claim, commissurotomy patients – having no direct commissural connections between the forebrain hemispheres – suffer in memory recall and recognition of word lists (e.g., Cronin-Golomb et al., 1996). Also, tasks that foster inter- as opposed to intra-hemispheric processing improve performance in neurotypical individuals (e.g., by reducing false memories; see Bergert, 2013; Christman et al., 2004), and bimanual (i.e., interhemispheric) coordination abilities are positively related to episodic-memory recall (Lyle et al., 2017). Consequently, systematic differences in the strength of the interhemispheric connectivity have been considered a candidate mechanism, attributing the improved performance in MH relative to cRH groups to a better connectivity in MH (e.g., Prichard et al., 2013). Evidence for this notion is typically taken from studies in which a bigger corpus callosum was found in MH (e.g., M. Habib et al., 1991; Luders et al., 2010; Witelson, 1989), although the results of a recent meta-analysis (Westerhausen & Papadatou-Pastou, 2022) and a large-scale study (Raaf & Westerhausen, 2023) did not confirm these differences. Nevertheless, differences in corpus callosum microstructure assessed with diffusion imaging as reported by McKay et al. (2017) or differences in functional connectivity such as transfer time (e.g., Bernard et al., 2011; Davidson & Tremblay, 2013; Kourtis et al., 2014), may still support the basic assumption of a stronger interhemispheric connectivity in MH.

Our primary aim in the present study was to revisit past findings of memory differences between MH and cRH in incidental learning by attempting a conceptual replication of the findings reported in Christman and Butler’s (2011) Experiment 1. We selected this study because it was one of the first to show handedness-related differences in incidental learning of verbal material. This finding is of special theoretical importance because, if confirmed, it supports the notion that the MH advantage originates in “built-in” automatic processing differences rather than in secondary differences in the conscious utilization of higher-order control or mediator strategies such as verbal associations or active imagery (Richardson, 1998). Building upon the level-of-processing theory (Craik & Lockhart, 1972; Craik & Tulving, 1975), Christman and Butler (2011) implemented a between-subject design with three incidental encoding conditions in which participants were asked to decide whether given word stimuli were (a) printed in upper or lower case letters (structural condition); (b) rhymed with a separate target word (phonemic); (c) fit into a given sentence (semantic); or in an intentional condition, participants were (d) instructed to study each word for a later memory test. Using hand preference assessed with a version of the Edinburgh Inventory (EHI, Oldfield, 1971), the authors divided each of the four groups into cRH and MH by using the overall median value of 80 of the EHI laterality quotient (LQ; range: −100 to 100, reflecting consistent left and right-hand preference, respectively) as cut-offs for the classification. Then, the authors compared the number of correctly recalled words (hits) between the cRH and MH across the four encoding conditions. Their main finding was a significant main effect of “handedness” (MH > cRH) which was qualified by an also significant statistical interaction with the “condition” factor. The post-hoc pair-wise comparisons revealed that the MH group had a higher number of hits than the cRH group both in the semantic incidental condition (Cohen’s *d* = .91) and the intentional encoding condition (*d* = 0.69), while this group difference was not significant in the two other incidental encoding conditions (phonemic: *d* = 0.11; structural: *d* = −0.09). Thus, Christman and Butler (2011) observed a MH advantage in incidental learning only when “deep“ semantic processing was required, but not under conditions of shallow processing.

We aimed to conceptually replicate Christman and Butler’s (2011) findings in the present pre-registered study (https://osf.io/qu4vh, Westerhausen & Johansen, 2022). Of note, there were three main deviations in our study design when compared with the original study. Firstly, for simplicity, we implemented only the “phonemic” and “semantic” encoding conditions for which we expected the interaction with handedness to be strongest. Secondly, we selected a within-subject rather than between-subject design to improve statistical power. Thirdly, we opted for a recognition task as opposed to a free-recall retrieval as recognition is better suited for separating overall response biases from the sensitivity of the memory retrieval (Lockhart, 2000) and a recognition test was used in the original level of processing studies (Craik & Tulving, 1975). Based on Christman and Butler’s (2011) findings, we predicted a significant interaction between the Encoding Condition and Hand preference Group, which was supposed to reflect a difference in the number of correctly recalled words in favor of MH over cRH for the semantic encoding condition, and no difference between the two groups for words processed in the phonemic condition. Our secondary objective was to obtain a more precise measure of the underlying population effect size, for which we collected additional data that we analyzed alone and in combination with data from the first replication sample.

## Method

### Ethical Considerations

The research protocol for this study received ethical approval from the local institutional review board of the Department of Psychology at the University of Oslo, Norway (Ref. number: 24818591). All participants provided informed consent to participate in the study.

### Participants

Our data collection for the first replication sample took place in February 2023. We first involved 60 participants, reduced to 49, after applying the exclusion criteria, as preregistered (Westerhausen & Johansen, 2022). That is, we excluded: (a) non-native speakers of Norwegian; (b) consistent left handers (defined as LQ ≤ −80; following the original study); and (c) participants with a false-alarm rate above .50 (see section *Memory Assessment* below). These 49 participants’ (25 female, 24 male) had a mean age of 26.0 years (standard deviation or *SD* = 10.4 years; ranging from 19 to 57 years). Applying the same criterion used by Christman and Butler (2011), we divided this sample into groups of 26 cRH (i.e., LQ >= −80) and 23 MH participants (see *Hand Preference Assessment* for details).

Of note, we based this sample size on an a priori power analysis in which we assumed the effect size for the interaction of “encoding” and “handedness” from findings in the original publication. That is, we extracted the mean values and standard errors from Figure 1 in Christman and Butler (2011, p. 19) for the relevant conditions (“phonemic” and “semantic”), and determined the effect size using the online analysis of variance (ANOVA) power app (https://shiny.ieis.tue.nl/anova_power/; Lakens & Caldwell, 2019). As documented in Supplement Section A (Figures S1 to S3, Table S1), the effect size of the interaction was determined as *η*_*p*_^*2*^ = .05. Of note, although the original study used a between-subject design on the factor “Condition,” we considered this effect size an appropriate estimation of a population effect when using a within-design, as in the present study. Using GPower software (Version 3.1.9.2.; Faul et al., 2009) and this effect size, we determined that statistical power of .80 (using alpha of 5%) would be achieved with a total sample size of 40 (i.e. 20 per group).

**Figure 1.**
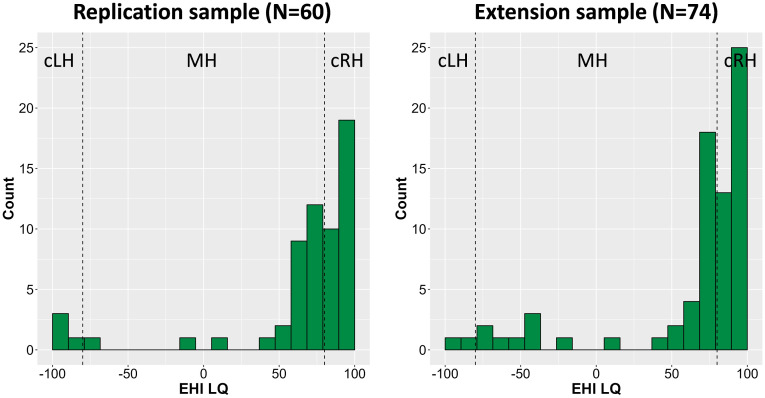
Distribution of the Laterality Quotient (LQ) Obtained with the Edinburgh Handedness Inventory in the Two Samples Before Data Exclusion. *Notes*. The LQ can reach from −100 to 100 for strong left- and right-hand preference, respectively. Following the approach chosen by Christman and Butler (2011), participants with an LQ of 80 and above (indicated by dotted line on the right side of each graph) were classified as consistent right-handers (cRH), and participants with a score between −80 and 80 as mixed handers (MH). Participants with an LQ of −80 and below were classified as consistent left handers (cLH), which were excluded from the main analyses.

The second replication-extension sample included data from 74 participants who were tested both before the registration (pilot) and after the conclusion of the replication study. Applying the same criteria as above, the remaining 65 participants (49 female, 14 male, and 2 self-classified as “other”) had a mean age of 23.4 years (*SD* = 4.4 years; ranging from 19 to 38 years). We divided this sample into a group of 34 cRH and 31 MH using the same criteria as before.

### Hand Preference Assessment

We assessed handedness using a Norwegian version of the EHI which asks for the same ten manual activities (e.g., writing, throwing) as the original version of the questionnaire (Oldfield, 1971). However, the Norwegian version utilizes a five-point scale response format (levels: “always left,” “mostly left,” “both equally often,” “mostly right,” and “always right;” cf. Edlin et al., 2015). As the response format might potentially affect the classification of participants into handedness groups (Papadatou-Pastou et al., 2013) it is important to note that Christman and Butler (2011) may have used a comparable five-point response format judging from Christman’s other publications (e.g., Christman et al., 2008). Our participants’ answers were scored from −2 (always left) to 2 (always right) on each EHI item. The scores were then summed across all ten items and multiplied by five to produce a LQ that ranged from −100 for a strong left-hand preference to +100 for a strong right-hand preference. In our initial replication sample, the LQ had a mean score of 63.2 (*SD* = 50.2) with a median value of 75, before the exclusion of participants. In our second sample, participants had an LQ average of 62.5 (*SD* = 51.2) with a median value of 80. As shown Figure 1, in both samples the distributions of the LQ followed the J-shaped curve that is expected for hand preference in the general population (Ocklenburg & Güntürkün, 2018).

### Memory Assessment

Our experimental paradigm followed that of Christman and Butler (2011) which, in turn, was based on Craik and Tulving (1975). However, we implemented only the “phonemic” and “semantic” (or “sentence”) incidental encoding conditions, omitting the “structural” and intentional conditions, since the handedness effect appeared in the original study in the step from the phonemic to the semantic level of processing. The encoding phase consisted of four blocks of each twelve neutral Norwegian nouns (such as “radio”, “ring”, “melon”). In each two of the blocks, participants were asked to judge the word based its phonemic (blocks 1 and 4) and semantic properties (blocks 2 and 3), respectively. In the “phonemic” encoding condition, participants judged whether the target word rhymed with another word provided (e.g. “*Does the word rhyme with “swing?”*) and respond by ticking a “*yes*” or “*no*” response box. In the “semantic” condition, participants evaluated whether the meaning of the target word fit into a given sentence (e.g., “*Thea is eating ______ for breakfast.*”). Again, participants provided only yes/no answers. The stimulus presentation method relied on Nettskjema, an online questionnaire platform provided by the University of Oslo. The word stimuli of one block were all presented at the same time and there were no presentation-time restrictions. That is, participants controlled the progression of the experiment themselves by pressing the button “continue.”

The retrieval phase consisted of a free-choice recognition test (Lockhart, 2000) following the format used by Craik and Tulving (1975). Participants were confronted with a list of 96 words, of which 48 were old (target) words, seen during encoding phase, and 48 words are new (distractor) words, presented in a pseudo-randomized order. Participants were tasked with indicating which words had been seen before by ticking “*yes*” or “*no*” boxes. All words were presented simultaneously in a scrollable list via Nettskjema, with ticking boxes behind each word. There were no time restrictions, but participants had to complete all 96 words before they could continue. We determined the number of correctly recognized targets (hits, *h*_*n*_) for both encoding conditions, and we calculated the proportion of false positive “recognitions” to the distractors (expressed as the false-alarm rate). In addition, to account for the sensitivity in distinguishing old and new items, we also determined *d'* for both encoding conditions (Lockhart, 2000; Stanislaw & Todorov, 1999) by subtracting the z-value of the false alarm rate from the z-value of the respective hit rate (the probability of selecting “yes” for old items). As *d'* can only be calculated when rates are neither 0 nor 1, 0 was replaced with 0.001 and 1 with 0.999 before calculation of *d’*. Table 1 shows the descriptive statistic of the dependent variables (*h*_*n*_, *d’*) s in the two samples.

**Table 1.** Descriptive Statistics of the Dependant Variables Split for the Two Encoding Conditions and the Two Sample (Irrespective of Handedness).

Condition	Variable	Replication sample (*N* = 49)		Extension sample (*N* = 65)	
		Mean	*SD* ^a^	Mean	*SD*
Phonemic	Number of hits (*h*_*n*_)	13.12	4.32	14.33	4.08
	Sensitivity (*d'*)	1.59	0.65	1.72	0.61
Semantic	Number of hits (*h*_*n*_)	21.29	2.24	21.66	1.73
	Sensitivity (*d'*)	2.85	0.98	2.81	0.65
Combined^b^	False alarm (rate)	0.11	0.10	0.10	0.07

*Note*: ^a^standard deviation.

^b^the recognition test took place simultaneously for the two encoding conditions so that false alarm could not be separated between the conditions.

### Procedure

After providing general instructions to participants, we began the encoding phase by successively presenting all four blocks. Then, participants completed the EHI questionnaire, which also served as a distractor task to minimize possible recency effects for the following surprise recognition test (i.e. the retrieval phase, see above). Collectively, these procedures took approximately 10 minutes.

### Statistical Analysis

Inferential statistical analyses involved two-factorial mixed-effect analyses of variance (ANOVA) with the between factor Handedness Group (cRH vs. MH) and the within-factor Encoding Condition. In keeping with the procedure in the original publication, we first used the number of correct identifications (*h*_*n*_) as the dependent variable. However, as we were concerned about potential response biases, we repeated the analysis using *d'* as the dependent variable. In both cases, the interaction was the effect of interest. We repeated these analyses three times, using data from (a) the replication sample, (b) the second, extension sample, and (c) the combined sample. We included the analysis of the combined sample to increase the sensitivity of our analyses (i.e., test power) for detecting effect sizes smaller than those reported by Christman and Butler (2011). All analyses were done in R, and the scripts and raw data are available via the accompanying OSF platform (https://osf.io/gb4fc/). We expressed effect sizes as partial eta squared (*η*_*p*_^*2*^*)* for main and interaction effects of the ANOVAs or as Cohen’s *d* for pairwise comparisons. We used an uncorrected alpha threshold of 5% as the criterion for all analyses, matching the ones used for the a priori statistical power calculation.

## Results

### Initial Replication Analyses (as Registered)

**Figure 2.**
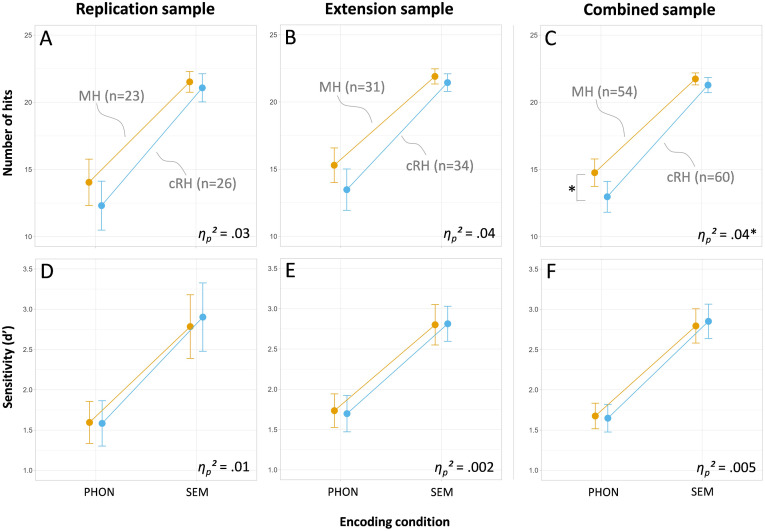
Mean and 95%-Confidence Limits for the Six Factorial Analyses (Split by Sample and Dependent Variable) with the Factors of Handedness and Encoding Condition (PHON = Phonemic, SEM = Semantic). *Notes.* Data from mixed-handed participants (MH) are printed in orange while data from consistent right handers (cRH) are printed in light blue. The partial eta square (*η*_*p*_^*2*^) values provided in each graph represents the size of the interaction effect. Asterisks (*) indicate significant interaction effects or post-hoc comparisons at *p* < .05; *n* denotes the number of participants in each subsample.

Using the *h*_*n*_ as the dependent measure, neither the main effect of Handedness Group (*F*_*1,47*_ = 1.70, *p* = .20, *η*_*p*_^*2*^ = .04) nor the interaction effect of Group and Condition were significant (*F*_*1,47*_ = 1.62, *p* = .21, *η*_*p*_^*2*^ = .03; see also Figure 2(A)). There was a significant main effect of Encoding Condition (*F*_*1,47*_ = 256.49, *p* < .001, *η*_*p*_^*2*^ = .85), indicating that participants showed a higher word recognition after semantic versus phonemic encoding. Comparably, using *d'*, again a significant main effect of Encoding Condition (*F*_*1,47*_ = 199.35, *p* < .001, *η*_*p*_^*2*^ = .81) was found, while neither the main effect of Handedness Group (*F*_*1,47*_ = 0.06, *p* = .81, *η*_*p*_^*2*^ = .001) nor the interaction effect of Group and Condition (*F*_*1,47*_ = 0.53, *p* = .47, *η*_*p*_^*2*^ = .01) were significant (see Figure 2(D)).

### Analysis of the Replication-Extension Sample

Analyzing *h*_*n*_ in the extension sample, the results were comparable as in the initial sample. Neither the main effect of Handedness Group (*F*_*1,63*_ = 3.32, *p* = .07, *η*_*p*_^*2*^ = .05) nor the interaction of Handedness Group and Encoding Condition were significant (*F*_*1,63*_ = 2.36, *p* = .13, *η*_*p*_^*2*^ = .04; see Figure 2(B)). The main effect of Encoding Condition was significant (*F*_*1,63*_ = 272.15, *p* < .001, *η*_*p*_^*2*^ = .81), reflecting higher recognition after semantic versus phonemic encoding. Again, the ANOVA using *d'* as the dependent measure only found a main effect of Encoding Condition (*F*_*1,63*_ = 219.74, *p* < .001, *η*_*p*_^*2*^ = .78), while neither the main effect of Handedness Group (*F*_*1,63*_ = 0.01, *p* = .93, *η*_*p*_^*2*^ < .001) nor the interaction of Group and Condition (*F*_*1,63*_ = 0.11, *p* = .74, *η*_*p*_^*2*^ = .002) were significant (see Figure 2(E)).

### Analysis of the Combined Samples

Combining the initial replication and the replication-extension samples, the ANOVA using *h*_*n*_ as the dependent measure revealed a significant main effect of Handedness Group (*F*_*1,112*_ = 4.92, *p* = .03, *η*_*p*_^*2*^ = .04), with a higher overall number of hits in the MH compared with the cRH group. This main effect was further qualified by a significant Group by Condition interaction (*F*_*1,112*_ = 4.03, *p* = .047, *η*_*p*_^*2*^ = .04; see Figure 2(C)) and by post-hoc t-tests indicating that the overall group differences in word recognition were driven by the phonemic (*t*_*112*_ = 2.31, *p* = .03, Cohen’s *d* = 0.44) rather than the semantic Encoding Condition (*t*_*112*_ = 1.35, *p* = .22, *d* = 0.23). The main effect of Encoding Condition was again significant (*F*_*1,112*_ = 529.11, *p* < .001, *η*_*p*_^*2*^ = .83). In contrast, when analyzing *d'* neither the main effect of Handedness Group (*F*_*1,112*_ = 0.02, *p* = .90, *η*_*p*_^*2*^ < .001) nor the interaction effect were significant (*F*_*1,112*_ = 0.55, *p* = .46, *η*_*p*_^*2*^ = .005; Figure 2(F)). The effect size for the group comparison between MH and cRH was *d* = 0.04 (confidence interval, *CI*_*95%*_: −0.32 to 0.41) in the phonemic and *d* = −0.07 (*CI*_*95%*_: −0.44 to 0.30) in the semantic encoding condition. The main effect of Encoding Condition was significant (*F*_*1,112*_ = 418.3, *p* < .001, *η*_*p*_^*2*^ = .79).

## Discussion

In our effort to replicate prior research on the presumed incidental learning advantage for MH versus cRH (Christman & Butler, 2011), neither in the replication nor in the extension sample did we find a significant interaction effect of Handedness Group by Encoding Condition, irrespective of whether we used the number of recognition hits or *d’* as the dependent measure. Only when we combined the two samples and thereby increased test power, did we find a statistically significant interaction effect for the number of hits. However, even in this instance the pattern of our results was reversed from the pattern reported by Christman and Butler (2011). While Christman and Butler (2011) found group differences in favor of MH over cRH in the phonemic rather than in the semantic encoding condition, we found the opposite. When we used *d’* rather than number of recognition hits as our dependent variable, the interaction effect was no longer significant, and the empirical effect size became negligible (explaining less than 0.5% of the variance). Importantly, using *d’* as opposed to the number of hits as dependent variable, appears more appropriate than using the hits as it accounts for potential report biases in the word-recognition test between participants and groups (e.g., Lockhart, 2000). We also found a small main effect of Handedness Group on the number of hits only after we combined both sample, which were again not able to detect when the more appropriate *d’* was used as dependent variable. Thus, our analyses neither supported an overall advantage in incidental learning nor a specific advantage in deep processing for MH over cRH participants.

Before further discussing the theoretical implications of our findings, we must consider to what degree our deviations from the original study design might have been relevant to these findings. Three major deviations require attention. Firstly, we selectively implemented the phonemic and semantic encoding condition, leaving out the intentional encoding and the shallow “structural” incidental encoding conditions used in the original study. As the crucial interaction effect reported by Christman and Butler (2011) includes all four conditions, one may wonder whether their discovery of an interaction effect was mainly driven by those conditions we omitted. Comparing the effect sizes of the semantic and phonemic conditions, this basis for our results is unlikely. The two effect sizes for the semantic and phonemic conditions appear to differ significantly from each other (i.e., the 95% confidence interval for Cohen’s *d* of the semantic condition is 0.29–1.52, not including the effect size of the phonemic condition) suggesting that the interaction term would also have been significant if only these two conditions had been used by Christman and Butler (2011). Additionally, the sample-size calculations we conducted through *a priori* power analysis were based only on data from the phonemic and semantic conditions (see Supplement Section A). Thus, the present research design should have had sufficient test power to replicate the original findings. Secondly, we implemented a within-rather than between-subject design used by Christman and Butler (2011). Here one might argue that the repeated-measurement design potentially fostered carry-over effects between the conditions. For example, once having performed a “deep” semantic analysis of the target words, participants may have not been able to go back to a “shallow” phonemic analysis. However, within designs have been traditionally and successfully used in comparable incidental learning studies (e.g., Challis et al., 1996; Craik & Tulving, 1975; Gardiner et al., 1996). Also, we found the expected level-of-processing effect with a large effect size (*η*_*p*_^*2*^ of .78 and .85 in our two samples) supporting that our within-subject design choice evoked the memory-enhancement effect that was predicted to be associated with semantic processing. Thirdly, we opted for a recognition test in the retrieval phase, rather than the free-recall test used by Christman and Butler (2011), as we considered recognition memory better suited to separate overall response biases from the sensitivity of the memory-retrieval process, and as it was used in the seminal study (Craik & Tulving, 1975). While some have suggested that a recognition test is less sensitive to detecting a MH advantage than a recall test (Propper & Christman, 2004), several others found handedness-related memory differences when using a recognition test (e.g., Chu et al., 2012; Lyle et al., 2012; McDowell et al., 2015; or as trend in Lyle, Logan, & Roediger, 2008). Thus, there is no evidence for the belief that using a recognition instead of a free-recall test would have systematically affected our results.

Our study failed to replicate the findings by Christman and Butler (2011) and suggests no MH advantage over cRH in incidental learning of word lists. Analyzing the data of our two samples combined, which provides the highest statistical power, the group differences in *d'* were close to zero in both the phonemic and the semantic condition, and substantial population effects (of a Cohen’s *d* of 0.4 and above, considering the confidence intervals) can be excluded with confidence. Thus, we feel encouraged to conclude that handedness-related differences in word recognition after incidental learning are negligible. However, our null finding deserves discussion regarding the modality of the memory content as well as the type of learning required. That is, some previous studies that found handedness effects for incidental learning strongly encouraged visual encoding by using pictural stories as stimulus material in eyewitness memory (Lyle, 2018; Lyle & Jacobs, 2010) or real-life content in autobiographical memory (Propper et al., 2005), while we focused on verbal encoding of word lists. Visual encoding – as initiated by visual stimulus material or through visual imagery – is typically associated with better memory recall than verbal encoding (Paivio, 1969, 1989). The superiority of visual encoding is thought to be related to the recruitment of visual-spatial abilities of the right hemisphere in addition to the verbal (typically) left hemisphere, emphasizing the role of interhemispheric connectivity during visual encoding (Liu et al., 2022; Paivio, 1989; Seamon & Gazzaniga, 1973). For example, patients with no corpus callosum (after commissurotomy) have seemed not to benefit from the instruction to visually imagine the to-be-learned words (Gazzaniga et al., 1975). Thus, one might speculate that, in past research, MH participants benefitted more than cRH participants from visual encoding due to their stronger interhemispheric connectivity (e.g., Bernard et al., 2011; Davidson & Tremblay, 2013; Kourtis et al., 2014; Lyle et al., 2017), resulting in the MH over cRH advantage for visual but not for verbal stimulus material. However, using intentional rather than incidental learning paradigms, also for verbal/word stimuli a MH advantage was frequently found (e.g., Christman & Butler, 2011; Chu et al., 2012; Propper et al., 2005; Propper et al., 2017). Thus, a difference in the stimulus material cannot be the main factor explaining our finding of no handedness-group difference. It can be assumed, however, that the intention to remember, as compared with incidental learning, motivates participants to engage in additional control processes during the encoding phase such as verbal associations or visual imagery (Craik, 2002; Richardson, 1998) increasing the binding of each word item into the context and increasing a likelihood for retrieval (Popov & Dames, 2023). Thus, one might predict that for verbal stimulus materials intentional memory mediation strategies are required to produce the MH advantage (e.g., by engaging right hemispheric processing i.e. automatically engaged when processing visual material). However, this interpretation is highly speculative and requires further empirical support before it can be accepted.

### Limitations and Directions for Further Research

One limitation, a feature of this field of research including the present study, is that the criterion for subdividing the sample with respect to hand preference consistency has been somewhat arbitrary (Hardie & Wright, 2014), and EHI LQ thresholds have varied across experiments, with different investigators using cut-offs of 75 (e.g., Prichard & Christman, 2017; Propper & Christman, 2004), 80 (e.g., Christman & Butler, 2011; M. Habib et al., 1991; Lyle, 2018), 85 (e.g., Propper et al., 2005), 95 (e.g., Lyle, McCabe, & Roediger III, 2008) or 100 (McDowell et al., 2016; Welcome et al., 2009) to separate cRH from MH participants. While some authors justified their criterion by stating that the sample median LQ was used as the threshold (e.g., Lyle, McCabe, & Roediger III, 2008; Propper et al., 2017), these differences between studies might explain some inconsistency in the results. In the present study we used a threshold of 80 and above to define cRH, applying numerically the same criterion as the one used by Christman and Butler (2011). Nevertheless, as we were curious as to whether choosing a different threshold value would alter our outcome, we conducted a series of exploratory analyses by systematically varying the cut-off criterion for this classification along the LQ continuum, including all previously used cut-offs. None of these substituted cut-offs (presented in Supplement Section B, Table S2) suggested any group difference in word recognition between individuals with consistent versus mixed-hand preference.

To be complete, we should mention that several authors reported differences in memory performance based on hand-preference direction (left vs. right handed) rather than the consistency (e.g., Jones & Martin, 1997; Lindell, 2023; Martin & Jones, 1999; McDowell et al., 2015; Piper et al., 2011). For example, Lindell (2023) found that left handers outperformed both mixed and right handers in immediate and delayed recall on the Rey Complex Figure Test, assessing visual memory. To test for a possible effect of handedness direction, we performed an additional exploratory analysis comparing individuals above and below an LQ of 0, to separate right and left handers, respectively. These results, using *d’* as the dependent variable, yielded neither a significant main effect of handedness preference nor a significant interaction effect (see Supplement Section C, Figure S4). Thus, we were not able to confirm an effect of handedness direction on incidental memory.

While the MH memory advantage appears well replicated for intentional learning (Prichard et al., 2013, 2023) our findings question this advantage for incidental learning. However, we used words as stimulus material which likely were subject to verbal encoding processes and we cannot exclude handedness-related memory differences resulting from non-verbal encoding. As outlined in the previous section, some investigators suggested that encouraging visual encoding can reveal handedness-related differences in memory also for incidental learning (Lyle, 2018; Lyle & Jacobs, 2010; Propper et al., 2005). These researchers relied on complex stimulus material, like slide-shows in eye-witness memory tasks, requiring a more elaborative processing during encoding. Thus, future investigators should test whether the MH memory advantage can also be found in tasks testing visual encoding of simple stimuli. The use of visual stimuli that are difficult to verbalize (see e.g., Silverberg & Buchanan, 2005) might be of particular interest in this approach.

## Conclusion

In summary, while prior research has suggested that there are systematic differences in memory performance between individuals with MH and cRH (Prichard et al., 2013, 2023), we were not able to replicate the advantage of MH over cRH individuals in incidental learning of verbal stimuli. We carefully planned our sample size and study design to achieve a statistically powerful replication of the original findings by Christman and Butler (2011), and we should have been able to detect effect sizes equal to or even smaller than those reported in the original study. As discussed, it seems also unlikely that small deviations in our experimental design relative to the original study would explain this outcome difference. Thus, we have no explanation for the difference in outcome between the two studies other than a random sampling bias. Based on our findings, we have no reason to believe that there is a relevant difference between MH over cRH participants in their verbal memory after incidental learning.

## Supplemental Material

Supplemental Material - No Incidental Memory Advantage for Participants With Mixed Handedness Compared to Those With Right Handedness: Conflicting Results From Earlier Research Supplemental Material for No Incidental Memory Advantage for Participants With Mixed Handedness Compared to Those With Right Handedness: Conflicting Results From Earlier Research by Henriette Johansen, Emilie H. Rusten, and René Westerhausen in Perceptual and Motor Skills
