# Supplementary material for: No Incidental Memory Advantage for Mixed Handed vs. Consistent Right Handed Participants: Conflicting Results From Earlier Research
Source: Percept Mot Skills. 2024 Oct 12;131(6):2049–68. doi: 10.1177/00315125241291266 (PMC11575099; doi:10.1177/00315125241291266)
Supplement: Supplemental Material - No Incidental Memory Advantage for Participants With Mixed Handedness Compared to Those With Right Handedness: Conflicting Results From Earlier Research [file sj-pdf-1-pms-10.1177_00315125241291266.pdf]

Supplement to manuscript: "*No advantage in incidental memory for mixed handers over consistent right handers. An unsuccessful conceptual replication*."

A. Effect size extraction and *a priori* power analysis – Figures S1 to S3, Tables S1

The following steps were performed for planning the optimal sample size for the replication experiment. Firstly, as the relevant mean values were not provided by Christman and Butler (2011), we used the WebPlotDigitizer (https://automeris.io/WebPlotDigitizer/) application to extract the mean values and error bar height (mean + standard error) for the number of correctly recalled items in phonemic and semantic encoding condition from Fig. 1 (p. 19). The following screenshot (Fig. S1) documents the procedure, and Table S1 shows the extracted values.

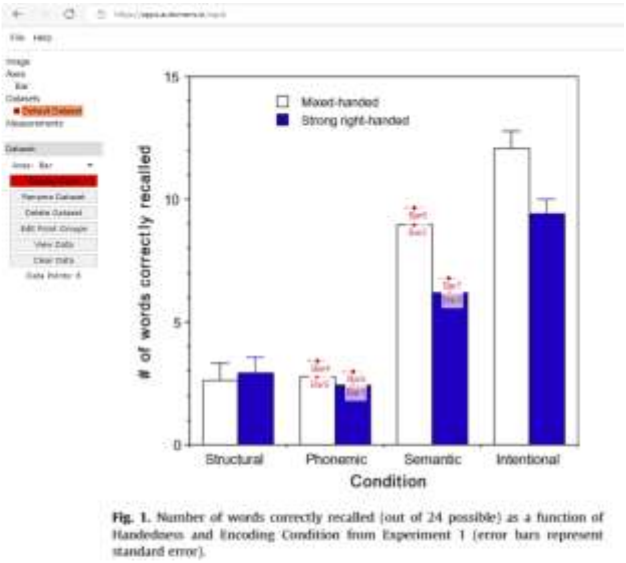

Fig. S1. Screenshot of the data extraction procedure using the WebPlotDigitizer

Table S1. Extracted values and calculated standard error (se) and standard deviation (sd), respectively

		Reading		Calculations		
Condition	Group	Mean	mean+se	se	n	SD
Phonemic	MH	2.77	3.42	0.64	22	3.02
	cRH	2.44	2.99	0.55	26	2.80
Semantic	MH	8.96	9.65	0.69	18	2.94
	cRH	6.21	6.78	0.57	30	3.14

Notes.

The means and standard deviations shown in Table 1 were then entered into the anova_power application (https://shiny.ieis.tue.nl/anova_power/; Lakens and Caldwell (2019)) to determine the effect size. Figure S2 shows a screenshot of the output.

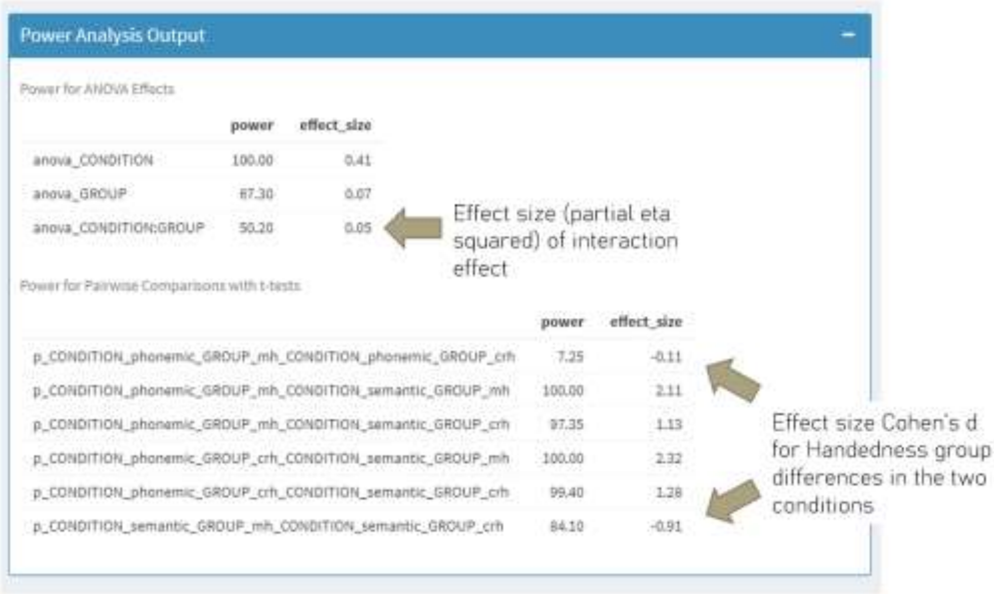

Power Analysis Output

Power for ANOVA Effects

	power	effect_size
anova_CONDITION	100.00	0.41
anova_GROUP	67.30	0.07
anova_CONDITION:GROUP	50.20	0.05

Power for Pairwise Comparisons with t-tests

	power	effect_size
p_CONDITION_phonemic_GROUP_mh_CONDITION_phonemic_GROUP_crh	7.25	-0.11
p_CONDITION_phonemic_GROUP_mh_CONDITION_semantic_GROUP_mh	100.00	2.11
p_CONDITION_phonemic_GROUP_mh_CONDITION_semantic_GROUP_crh	97.35	1.13
p_CONDITION_phonemic_GROUP_crh_CONDITION_semantic_GROUP_mh	100.00	2.32
p_CONDITION_phonemic_GROUP_crh_CONDITION_semantic_GROUP_crh	99.40	1.28
p_CONDITION_semantic_GROUP_mh_CONDITION_semantic_GROUP_crh	84.10	-0.91

Fig. S2. Effect size determination for the original interaction and the pairwise comparisons. The columns named “power” provide the observed power, i.e. the test power the original study has (if repeated) to find an effect of the originally found effect size.

Of note, the interaction effect size is calculated for the 2 x 2 design (leaving out two levels from the factor condition) reported by Christman and Butler (2011). Also, the present study used a within (repeated measures) design for the factor Encoding Condition rather than a between design used by the original study. Nevertheless, we used the above determined effect size of a partial eta squared (η_p^2) of 0.05 as best guess for what to expect in the population. We consider this a conservative approach as effects including repeated-measure designs can be expected to be larger, as participant related error variance is accounted for during the analysis.

The sample size calculations were then conducted using the GPower software (Faul, Erdfelder, Buchner, & Lang, 2009) as shown in the screenshot presented in Fig. S3. The results indicate that a sample of N = 40 (i.e. 20 in each group) is required to secure a test power of .80 for a population effect size of $\eta_p^2 = 0.05$ (or Cohen's $f = 0.229$)

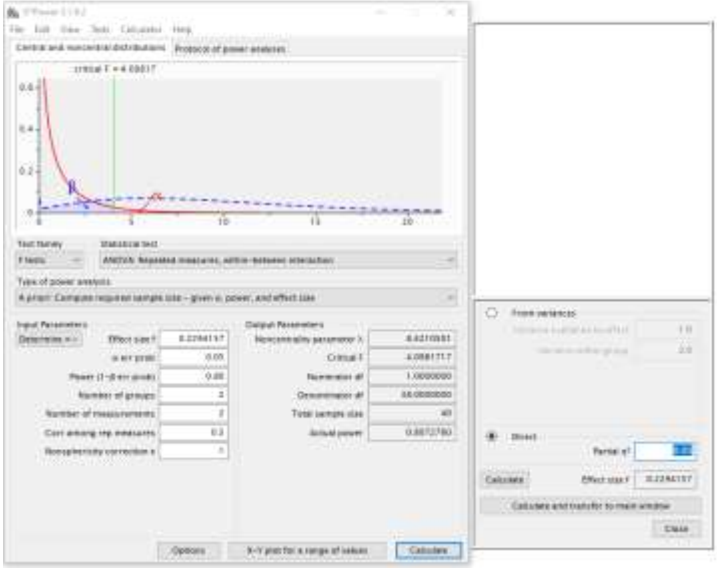

Fig. S3. A priori power analysis using GPower.

B. Varying the laterality quotient (LQ) threshold for separating consistent (cH) from mixed handers (MH) – Table S2

Using a specific LQ-threshold (e.g., LQ = 80 done in the presents study following Christman and Butler (2011)) to separate consistent right-handers cRH from MH is essentially arbitrary and various thresholds have been used in the literature. To examine how the use of divergent thresholds might affect the outcome we conducted an exploratory analysis using absolute value LQ-thresholds between 20 and 100 to separate a group of consistent handedness (cH) from MH. We used the absolute value to be able to evaluate the strength of hand preference independent from the direction. This also increases the sample size to N = 119 as formerly excluded consistent left handers (cLH) were now included. All analyses were set-up as two-factorial analyses of variance with the factors Handedness and Encoding condition (within factor). We used *d*' as dependent variable, as it appears more appropriate than using the number of hits when a recognition retrieval is used (Lockhart, 2000). As can be seen in Table S2, neither main effect of Handedness nor the interaction was significant for any of the analyses

Table S2. Effect of varying the LQ threshold (LQth) for defining handedness groups on the main effect of handedness (HD) and the interaction of HD and encoding condition (ENC) when analysing *d'* as dependent variable

	Sample size		Main effect HD			Interaction HD x ENC		
LQth[a]	MH	cH	$F_{1,117}$	p	η_p^2	$F_{1,117}$	p	η_p^2
20	3	116	2.75	0.10	0.02	2.33	0.13	0.02
30	4	115	1.91	0.17	0.02	1.61	0.21	0.01
40	4	115	1.91	0.17	0.02	1.61	0.21	0.01
50	9	110	0.35	0.56	<0.01	0.27	0.60	<0.01
60	12	107	1.38	0.24	0.01	3.20	0.08	0.03
70	24	95	1.05	0.31	0.01	2.98	0.09	0.03
75	38	81	0.70	0.41	<0.01	0.30	0.59	<0.01
80[b]	54	65	<0.01	0.97	<0.01	0.37	0.55	<0.01
85	57	62	0.34	0.56	<0.01	<0.01	0.94	<0.01
90	74	45	2.25	0.14	0.02	1.49	0.23	0.01
95	88	31	1.38	0.24	0.01	0.36	0.55	<0.01
100	101	18	3.29	0.07	0.03	0.32	0.57	<0.01

Notes. (a) participants with an absolute value of LQ of LQth and above were classified as consistent (cH), those below as mixed handers (MH); (b) this analysis is the same analysis as reported for the combined sample in the main analysis but includes the otherwise excluded consistent left handers in the cH group.

C. The effect of direction of handedness – Fig. S4

As outlined in the main text, several studies report memory differences comparing participants based on the direction (left vs right handers) rather than consistency of hand preference. To test for such an effect in the present sample, we repeated the analysis using an LQ = 0 as cut off to separate left handers (LH) from right handers (RH; no participant had LQ = 0). This procedure yielded 14 LH (11.8%) and 105 RH. The two-factorial analysis of variance did neither find a main of Handedness ($F_{1,117} = 0.34$, $p = .56$, $\eta_p^2 < .01$) nor an interaction of Handedness and Encoding Condition ($F_{1,117} < 0.01$, $p = .98$, $\eta_p^2 < .01$; see also Fig. S4 below). As for all other analyses, the main effect of Encoding condition was significant ($F_{1,117} = 184.66$, $p < .001$, $\eta_p^2 = .61$). However, the comparably low number of LH participants requires the null finding with respect to the direction of handedness to be replicated with larger samples of LH.

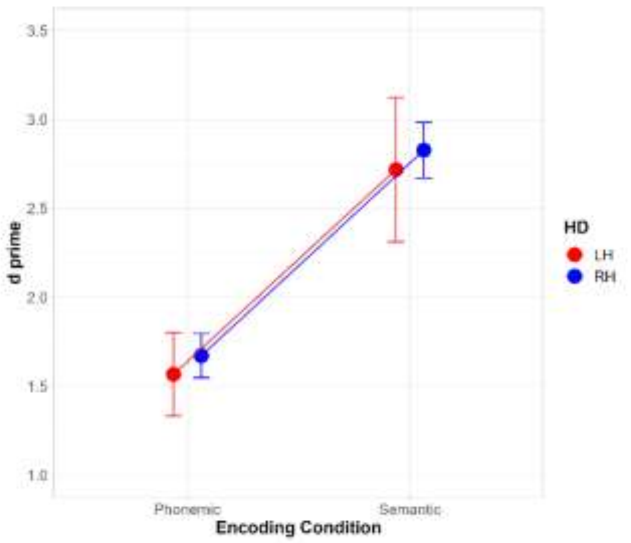

Fig. S4. Mean *d'* values (± 95% confidence intervals) for the interaction of handedness (left, LH, vs. right hander, RH) and Encoding Condition, and using. Neither the main effect of Handedness nor the interaction was significant in the two-factorial analysis of variance.

References

Christman, S. D., & Butler, M. (2011). Mixed-handedness advantages in episodic memory obtained under conditions of intentional learning extend to incidental learning. *Brain and cognition, 77*(1), 17-22.

Faul, F., Erdfelder, E., Buchner, A., & Lang, A.-G. (2009). Statistical power analyses using G* Power 3.1: Tests for correlation and regression analyses. *Behavior research methods, 41*(4), 1149-1160.

Lakens, D., & Caldwell, A. R. (2019). Simulation-Based Power-Analysis for Factorial ANOVA Designs. . doi:10.31234/osf.io/baxsf

Lockhart, R. S. (2000). Methods of memory research. In E. Tulving & F. I. Craik (Eds.), *The Oxford handbook of memory* (pp. 45-57). New York: Oxford University Press.
